# Quantitation of 6-, 8- and 10-Gingerols and 6-Shogaol in Human Plasma by High-Performance Liquid Chromatography with Electrochemical Detection

**Published:** 2010-09

**Authors:** Suzanna M. Zick, Mack T. Ruffin, Zora Djuric, Daniel Normolle, Dean E. Brenner

**Affiliations:** 1 *University of Michigan, Department of Family Medicine, 24 Frank Lloyd Wright Drive, P.O. Box 385, Lobby M, Ann Arbor, MI 48106, USA;*; 2 *University of Michigan, Department of Family Medicine, 1500 East Medical Center Drive, CCGC 6-303, Ann Arbor, MI 48109-0944, USA;*; 3 *University of Michigan, Department of Radiation Oncology, 1500 East Medical Center Drive, CCGC 6-303, Ann Arbor, MI 48109-0944, USA;*; 4 *University of Michigan, Department of Internal Medicine, 1500 East Medical Center Drive, CCGC 6-303, Ann Arbor, MI 48109-0944, USA*

**Keywords:** ginger, gingerols, shogaols, analytical methods, high performance liquid chromatography

## Abstract

*Zingiber officinale* is one of the most commonly used spices. We developed a method to determine the main pungent ginger constituents, 6-, 8- and 10-gingerols and 6-shogaol in human plasma. Quantitation was achieved using a reversed-phase C_18_ column using high-performance liquid chromatography with electrochemical detection. The assay was linear from 0.1 to 5.0 μg/mL. The within-day coefficients of variation for the assay at 5.0 μg/mL were ≤5% for all analytes. The recovery of all four analytes was ≥99% for at 5.0 μg/mL. The lower limit of quantitation was 0.1 μg/mL except for 10-gingerol which was 0.25 μg/mL. Currently, there is no analytical method for detecting pungent ginger constituents in human plasma. This HPLC method allows for the detection of all four of ginger’s pungent constituents simultaneously in a relatively short run time of 25 minutes. This method should be useful for determining plasma levels of 6-, 8-, 10-gingerol and 6-shogaol in phase I clinical trials.

## INTRODUCTION

The ginger root or rhizome (*Zingiber officinale* Roscoe, Zingiberaceae) is one of the most heavily consumed dietary substances in the world (1). Ginger was first cultivated in Asia, and has been used as a medicinal herb for at least 2,000 years (2). In Western herbal medicine, ginger is used as a circulatory stimulant, a cold and flu treatment, and a remedy for digestive disorders including dyspepsia, colic, nausea, vomiting, gastritis, and diarrhea (3).

In recent years, numerous promising human studies have explored the efficacy and safety of oral doses of ginger root as an anti-nausea agent and for relieving pain and swelling. Moreover, ginger has been investigated *in vitro* and in animal models for its cancer prevention (1), anti-inflammatory (4), and anti-diabetic (5) activities. These studies have increased the interest in possible medicinal benefits of ginger root and contributed to ginger being one of the top twenty dietary supplements sold in the United States (6).

Ginger contains approximately 1.0 to 2.5% pungent constituents (a non-volatile oily liquid consisting of homologous polyphenols) that give ginger its pungent or hot quality (7). The root’s pungent constituents are responsible for ginger’s anti-nausea and anti-inflammatory effects (8). In ginger, all of the pungent compounds contain the vanilly (4-hydroxy-3-methoxphenyl) moiety and a ketone functional group in their structures (9). Gingerols, paradols, zingerones, and shogaols are the main class of pungent or phenolic compounds in the root (10). Gingerols are the most abundant compounds in fresh roots and several gingerols of various chain-lengths (n6 to n10) are present in ginger with the most abundant being 6-gingerol. Shogaols, the dehydrated form of gingerols, are found in only small quantities in the fresh root and are mainly found in the dried and thermally treated roots with 6-shogaol being the most abundant (11).

Despite ginger being used in over 30 clinical trials in humans with over 2300 subjects (10), only a handful of studies in rats and our study in healthy volunteers (12) have examined the absorption, bioavailability, metabolites and elimination of ginger constituents. In rat studies, only two of the pungent compounds, 6-gingerol and zingerone, have been investigated, and in two of the rat studies 6-gingerol was administered as an intravenous bolus (13, 14), which is unlikely to be reflective of usual oral dosing. Moreover, until we conducted a study in healthy volunteers no pharmacokinetic studies have been conducted in humans nor had any studies in mammals or *in vitro* examined the other major pungent constituents, namely 8- and 10-gingerols and 6-shogaols.

One major limitation to conducting pharmacokinetic studies of pungent ginger constituents in humans is the current lack of an extraction procedure and analytical assay that could be used to detect various gingerols and shogaols in human plasma. The purpose of this paper is to present the development and validation of a high performance liquid chromatography (HPLC) assay with electrochemical detector for 6-, 8- and 10-gingerols and 6-shogaol from human plasma.

## EXPERIMENTAL METHODS

### Chemicals

**Figure 1:**
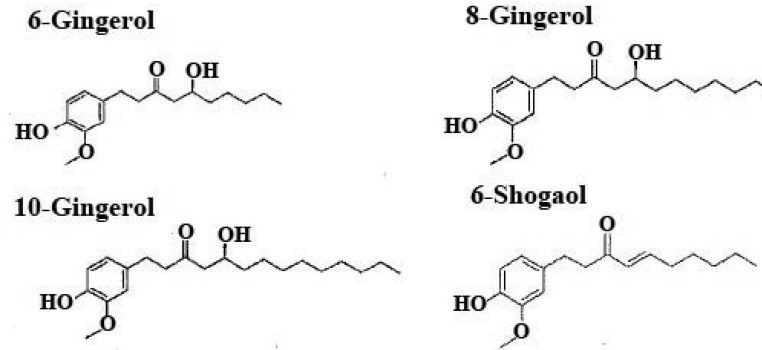
Chemical structures of 6-, 8- and 10-gingerols and 6-shogaol.

Six, 8, and 10-gingerols and 6-shogaol were purchased from Chromadex (Santa Ana, CA, USA: Catalog numbers ASB-00007164-005, ASB-00007163-005, ASB-00007162-005, ASB-00019211-005) (Figure 1). Standards were found to be >95% pure per HPLC analysis. Pelargonic acid vanillylamide (PAV) was obtained from Sigma (St. Louis, MO, USA) and is ≥ 97% pure. Acetonitrile, methanol, hexane and de-ionized water were all HPLC grade (Burdick & Jackson, Muskegon, MI, USA). HPLC grade ethyl acetate and ammonium acetate were purchased from Fisher Scientific (Pittsburgh, PA, USA). HPLC grade acetic acid was obtained from J.T. Baker (Phillipsburg, NJ, USA).

### Instrumentation and equipment

The HPLC system consisted of a refrigerated Waters 717 plus auto-sampler, 600E solvent controller and Waters in-line degasser AF (Waters, Milford, MA, USA), and an electrochemical Model 5600A CoulArray detector (Chelmsford, MA, USA). Chromatographic separation was accomplished using a Phenomenex Luna 4.6 mm × 250 mm, S-5 μm, C_18_ column that was coupled with a Phenomenex 4.0 mm × 20 mm, 5 μm C_18_ guard column (Phenomenex, Torrance, CA, USA).

The determination of extinction coefficients was obtained using a Hewlett Packard 8452A Diode Array Spectrophotometer (Hewlett Packard, Palo Alto, CA).

### Standards and quality control sample preparation

Oily 6-gingerol or 8- and 10-gingerol and 6-shogaol were weighed and dissolved in 1.0 mL methanol to obtain a stock standard of each at 5.0 mg/mL. A working standard was prepared by combining all four of the stock standards (6, 8 and 10-gingerol and 6-shogaol) together to achieve a concentration of 500 μg/mL for each compound, from which six other working standards were prepared by dilution and stored at -35°C.

Validation of the assay was performed by spiking plasma with known amounts of standards. Blood samples for validation were collected in heparinized tubes from human volunteers who were participating in the *in vivo* experiment described below and had not consumed ginger within the past 72 hours. There was no detectable 6-, 8-, or 10-gingerols or 6-shogaol in this plasma. Human plasma was stored at -70°C until used for the gingerols/shogaol assay.

PAV, a synthetic analogue of capsaicin, was the internal standard. PAV has been previously used as a standard for HPLC separation of gingerols and shogaols (15). The internal standard was prepared by dissolving 10 mg of powdered PAV in 1.0 mL of methanol for a final concentration of 10 mg/mL (stock standard). A working internal standard was prepared in methanol at a concentration of 100 μg/mL.

### Analytical procedure and sample preparation

Plasma samples (490 μL) were spiked with 10 μL of various concentrations of combined working standards (125.0, 50.0, 25.0, 12.5 & 5.0 μg/mL) and vortexed (Vortex-Genie: Fisher Scientific, Pittsburgh, PA, USA) for 20 s at 1,700 g. Ten μL of internal standard, (100 μg/mL) were then added and vortexed again for 20 s at 1,700 g. The samples were diluted at a 1:1 ratio with HPLC grade water and vortexed again for 20 s at 1,700 g. The samples were diluted with water in a 1:1 ratio to greatly increase the separation between lipid soluble and water soluble layers. Samples were then extracted with 2 mL ethyl acetate: hexane (1:1 v/v) and vortexed for 20 s three times for a total of 60 s at 1,700 g. After centrifugation for 10 minutes at 1,000 g, the upper organic layer was removed manually using a 5 ¾ inch Corning® disposable glass transfer pipette (Fisher Scientific: Pittsburgh, PA, USA) into clean 200 μL auto-sampler insert 2.0 mL tubes and dried under a stream of argon at room temperature. The samples were re-suspended in 60 μL of HPLC grade acetonitrile, vortexed for 20 s at 1,700 g, and 40 μL of HPLC grade water was added followed by vortexing for another 5 s at 1,700 g. Samples were then left in the dark at -4°C for at least 10 minutes to allow for through mixing. Samples were filtered through a 0.45 μm nylon filter (Sigma, St. Louis, MO, USA), located within a 1.7 mL Corning® microcentrifuge tube, with snap cap, by centrifugation for 5 minutes at 3,200 g. The filtered samples were then placed into 250 μL HPLC autosampler vials for HPLC quantification.

### HPLC analytical run

The HPLC method number 114.000 of the National Sanitation Foundation (NSF) was modified for analysis of plasma samples (15). 6-, 8- and 10-gingerols and 6-shogaol were separated and quantified by HPLC using electrochemical detection (EC) at 600, 550 and 500 mV. Reagent A was acetonitrile:water:ammonium acetate (59:39:2 v/v/v). Reagent B was 100% acetonitrile containing 20 mL of 1.0M ammonium acetate at pH4.5 (98:2 v/v). The pH of the ammonium acetate was brought to 4.5 with the addition of 1.0M acetic acid. The ammonium acetate was filtered, through 0.45 μm filter (Millipore, Bedford, MA, USA). An aliquot (20 μL) was injected into a reverse-phase C_18_ column and eluted with a gradient changing from reagent A (100%) to reagent B (100%) over the course of 15 minutes using a Waters #4 curve (concave). This was followed by 100% reagent B for 10 minutes and completed with a column wash of 100% reagent A for 10 minutes. The flow rate was 0.8 mL/minute.

### Methods for determining extraction efficiency and estimation of accuracy and precision

The standards for determining extraction efficiency were made in triplicate by adding internal standard (at a concentration of 10.00 μg/mL) and a combination of 6, 8, 10- gingerols and 6-shogaol at three different concentrations: high (5.00 μg/mL), medium (1.00 μg/mL) and low (0.25 μg/mL) to methanol. In addition, the same high, medium and low concentrations of 6-, 8-, and 10-gingerols and 6-shogaol were prepared in plasma along with internal standard (10.00 μg/mL). Five samples of spiked plasma at each concentration were made and extracted. All samples were analyzed in the same analytical run.

The extraction efficiency of the method was determined by comparing standard ginger solutions in methanol to the samples extracted from plasma matrices. Two calculations were used to obtain the extraction efficiency. First, the mean peak area from an extracted plasma sample was divided by the mean area of a methanol solution at the same concentration to give a “raw data percent recovery” using the addition of the internal standard. Second, 6-, 8- and 10-gingerols and 6-shogaol at each concentration (5.00, 1.00, 0.25 μg/mL) were quantified by peak area ratio (gingerols/shogaol analytes to internal standard) in all the samples, and the calculated amount in plasma divided by the known amount in methanol solutions.

The inter-assay accuracy and precision of the method was assessed using three different sample pools that contained combined 6-, 8-, 10-gingerols and 6-shogaol in the 5.00, 1.00 and 0.25 μg/mL concentrations and internal standard. Accuracy was determined for each analyte at each concentration (5.00, 1.00 and 0.25 μg/mL) separately on the four different days by determining the mean concentration and standard deviation (SD) of the five aliquots and dividing the SD by the mean concentration to determine the coefficient of variation (CV) (16).

The quantification of 6-, 8- and 10-gingerols and 6-shogaol was accomplished by peak area ratio (gingerols/shogaol analytes to internal standard) that was based on a standard curve prepared in plasma matrix. The standard curve was run in duplicate and covered six concentrations: 5.00, 2.50, 1.00, 0.50, 0.25 & 0.10 μg/mL. A line was fit using the least squares criterion for each of the four analytes (gingerols/shogaol) on the six different standard concentrations run (5.00 to 0.10 μg/mL) in each batch.

### Determining lower limits of quantitation and detection

Limits of detection in a plasma matrix for all ginger analytes were calculated using a 3:1 ratio of the height of the lowest detectable peak to the height of the largest baseline peak (baseline noise). Thus, a peak height of a ginger analyte three times higher than the highest baseline peaks would be considered the limit of detection for the assay. The concentration of the ginger analyte in a plasma matrix for the limit of detection was calculated by multiplying the height of the largest baseline peak by 3 (peak height of the limit of detection) and then using a ratio of the peak height of lowest concentration quantifiable for each ginger analyte (0.10 μg/mL for 6-, 8- gingerol and 6- shogaol and 0.25 μg/mL for 10-gingerol) per the following formula: (PH_LD_•C_LQ_)/PH_LQ_=C_LD_. Where PH_LD_=Peak height of the limit of detection, PH_LQ_=Peak Height of the limit of quantification, C_LQ_=Concentration of analyte at limit of quantification and C_LD_=Concentration of analyte at limit of determination.

## RESULTS

### Determining the extinction coefficients

The extinction coefficients for 6-, 8-, 10-gingerols and 6-shogaol were estimated using the diode array spectrophotometer. Five different concentrations (ranging from 5.0 to 0.5 μg/mL) of each analyte in methanol were scanned in triplicate and an average absorption calculated. The wavelength of maximal absorption was 282 nm for each compound, and at that wavelength, the extinction coefficients were 2530 M^-1^ for 6-gingerol, 2391 M^-1^ for 8-gingerol, 2182 M^-1^ for 10-gingerol and 2391 M^-1^ for 6-shogaol.

### HPLC Analysis

A linear relationship was identified for each of the four ginger plasma analytes ranging between the 0.10 μg/mL and 5.00 μg/mL. For each of the standard curves six different concentrations (5.00, 2.50, 1.00, 0.50, 0.25 & 0.10 μg/mL) were used to establish the linear range. Coefficients of determination (r^2^) ranged from 0.9854 to 0.9992 for all four analytes using concentrations determined by area ratios. Slopes and intercepts of the standard curves for all four analytes were similar across the four days of validation (Table 1).

**Table 1:** Characteristics and reproducibility of the 6-, 8-, and 10-gingerol and 6-shogaol plasma extraction method

Analyte in plasma	Concentration Range (μg/mL)	Day of assay	Slope	Intercept	Coefficient of determination (r^2^)

6-gingerol	0.1 to 5.0	1	0.30	0.01	0.9920
		2	0.37	0.02	0.9949
		3	0.35	0.02	0.9986
		4	0.35	0.02	0.9983
8-gingerol	0.1 to 5.0	1	0.21	0.02	0.9947
		2	0.17	0.02	0.9936
		3	0.18	0.02	0.9973
		4	0.17	0.01	0.9992
10-gingerol	0.1 to 5.0	1	0.07	0.00	0.9911
		2	0.05	0.00	0.9896
		3	0.06	0.00	0.9954
		4	0.05	0.00	0.9969
6-shogaol	0.1 to 5.0	1	0.24	0.03	0.9854
		2	0.17	0.00	0.9935
		3	0.17	0.02	0.9929
		4	0.17	0.01	0.9989

### Extraction efficiency

The extraction efficiency derived from raw data ranged from 60.4 to 104.8 % depending on the analyte and its concentration in plasma. Similarly, the extraction efficiency derived from area ratios ranged from 82.5 to 165.3%. Extraction efficiency based on both the raw data and area ratios for all four analytes at high, medium and low concentrations is presented in Table 2.

**Table 2:** Extraction efficiency for 6, 8 and 10-gingerol and 6- shogaol analytes

Analyte in plasma	Concentration (μg/mL)	Percent Recovery Based on Raw Data	Percent Recovery Based in Area Ratios

6-gingerol	5.00	69.5	113.0
	1.00	73.3	100.6
	0.25	64.1	87.6
8-gingerol	5.00	64.9	105.5
	1.00	76.5	105.1
	0.25	78.1	106.8
10-gingerol	5.00	100.8	165.3
	1.00	104.8	144.1
	0.25	102.3	139.8
6-shogaol	5.00	61.5	99.9
	1.00	66.3	91.1
	0.25	60.4	82.5

### Estimation of accuracy and precision

Low, medium and high concentrations of the ginger analytes were assayed across 4 different days to determine the accuracy and precision of the method. The intra-day values for the CV ranged from 1.5 to 10.7 % and the inter-day CV ranged from 1.0 to 11.5 % for all four analytes. The CVs are presented along with means and SDs in Table 3.

**Table 3:** Intra- and inter-assay variability for 6, 8, and 10-gingerol and 6-shogaol analyses

Analyte in plasma	Sample type	Calculated Concentration on (μg/mL)	Number of assays	Concentration (Mean and [SD]) (μg/mL)	Coefficient of Variation (%)	Day of analysis

6-gingerol	Plasma, high	5.000	5	5.06 (0.18)	3.6	1
		5.000	5	5.11 (0.29)	5.7	2
		5.000	5	4.95 (0.14)	2.8	3
		5.000	5	4.90 (0.28)	5.8	4
	Plasma, medium	1.000	5	0.87 (0.03)	2.9	1
		1.000	5	0.81 (0.02)	2.1	2
		1.000	5	0.84 (0.04)	4.6	3
		1.000	5	0.81 (0.02)	2.4	4
	Plasma, low	0.250	5	0.28 (0.03)	9.5	1
		0.250	5	0.16 (0.00)	2.3	2
		0.250	5	0.22 (0.01)	6.1	3
		0.250	5	0.25 (0.01)	3.7	4
8-gingerol	Plasma, high	5.000	5	4.96 (0.16)	3.3	1
		5.000	5	4.93 (0.42)	8.4	2
		5.000	5	4.92 (0.14)	2.8	3
		5.000	5	5.14 (0.24)	4.8	4
	Plasma, medium	1.000	5	0.78 (0.03)	4.1	1
		1.000	5	0.85 (0.06)	7.1	2
		1.000	5	0.79 (0.05)	5.8	3
		1.000	5	0.86 (0.06)	7.6	4
	Plasma, low	0.250	5	0.24 (0.00)	1.7	1
		0.250	5	0.24 (0.02)	6.6	2
		0.250	5	0.22 (0.02)	10.7	3
		0.250	5	0.25 (0.02)	7.5	4
10-gingerol	Plasma, high	5.000	5	5.20 (0.09)	1.8	1
		5.000	5	4.96 (0.29)	5.8	2
		5.000	5	5.12 (0.28)	5.5	3
		5.000	5	4.99 (0.48)	9.7	4
	Plasma, medium	1.000	5	1.03 (0.08)	8.1	1
		1.000	5	0.86 (0.01)	1.5	2
		1.000	5	1.05 (0.08)	7.9	3
		1.000	5	1.00 (0.09)	8.8	4
	Plasma, low	0.250	5	0.41 (0.03)	7.7	1
		0.250	5	0.48 (0.03)	6.5	2
		0.250	5	0.36 (0.02)	6.5	3
		0.250	5	0.57 (0.03)	4.8	4
6-shogaol	Plasma, high	5.000	5	5.11 (0.38)	7.4	1
		5.000	5	4.96 (0.29)	5.8	2
		5.000	5	4.95 (0.49)	10.0	3
		5.000	5	5.04 (0.48)	9.4	4
	Plasma, medium	1.000	5	0.79 (0.05)	6.7	1
		1.000	5	0.86 (0.01)	1.5	2
		1.000	5	0.81 (0.04)	5.1	3
		1.000	5	0.84 (0.03)	4.1	4
	Plasma, low	0.250	5	0.22 (0.00)	1.6	1
		0.250	5	0.23 (0.02)	9.8	2
		0.250	5	0.27 (0.02)	7.6	3
		0.250	5	0.26 (0.02)	7.4	4

### Limits of quantitation and limits of detection and specificity

The limit of quantification in human plasma using the electrochemical detector for 6-gingerol, 8- gingerol and 6-shogaol were 0.10 μg/mL while the limit of quantification was 0.25 μg/mL for 10-gingerol. The limit of detection in plasma using 3:1 ratio of the height of the lowest detectable peak to the height of the largest baseline peak (baseline noise) for 6-ginerol was 0.065 μg/mL, for 8-gingerol was 0.053 μg/mL, for 10-gingerol was 0.037 μg/mL and for 6-shogaol was 0.075 μg/mL.

There also were no peaks in healthy adult human plasma that overlapped with the gingerols and shogaol analytes and the internal standard after oral ginger intake. Moreover, the use of 3 electrochemical voltages yielded a distinct ratio of signals for each compound that helps confirm identity of the peak.

### *In vivo* experiment

To demonstrate the utility of the HPLC method in humans, a brief description of the results in a few participants taking ginger orally is presented. Full study results are published elsewhere (12). In brief, a single dose of 2.0 g of ginger extract was administered orally as eight 250 mg capsules to four healthy adult volunteers not taking any chronic medications or NSAIDS within 28 days of the blood draws. Blood was drawn from the participants at baseline, 15, 30, and 45 minutes as well as at 1, 2, 4, 6, 10, 24, 48 and 72 hours after ingestion of the ginger. All study procedures were administered at the University of Michigan General Clinical Research Center (GCRC) after the participant gave written, informed consent, and the study was approved by the University of Michigan Institutional Review Board. The ginger product used in this study was manufactured by Pure Encapsulations^®^ (Sudbury, MA) (batch #ZO/06006). Pure Encapsulation’s^®^ ginger (*Zingiber officinale*) powder is processed using Good Manufacturing Procedures (GMP). Each capsule contained 250 mg dry extract of ginger root [10:1 (v/v) extraction solvent (ethanol 50 %/water 50%): root] standardized to 15 mg (5%) of total gingerols. Based on HPLC analysis a 250 mg capsule of ginger extract contained 5.38 mg (2.15%) 6-gingerol, 1.80 mg (0.72%) 8-gingerol, 4.19 mg (1.78%) 10-gingerol, and 0.92 mg (0.37%) 6-shogaol. The University of Michigan Investigational Drug Service (UM IDS) placed 250 mg of the Pure Encapsulations^®^ ginger powder in size “0” red animal gelatin capsules made by Gallipot^®^ (17). Content of ginger analytes in the study medication were independently verified using appropriate high performance liquid chromatography methods (Integrated Biomolecule; Tuscon, AZ) as well as being verified by Pure Encapsulations.

**Figure 2:**
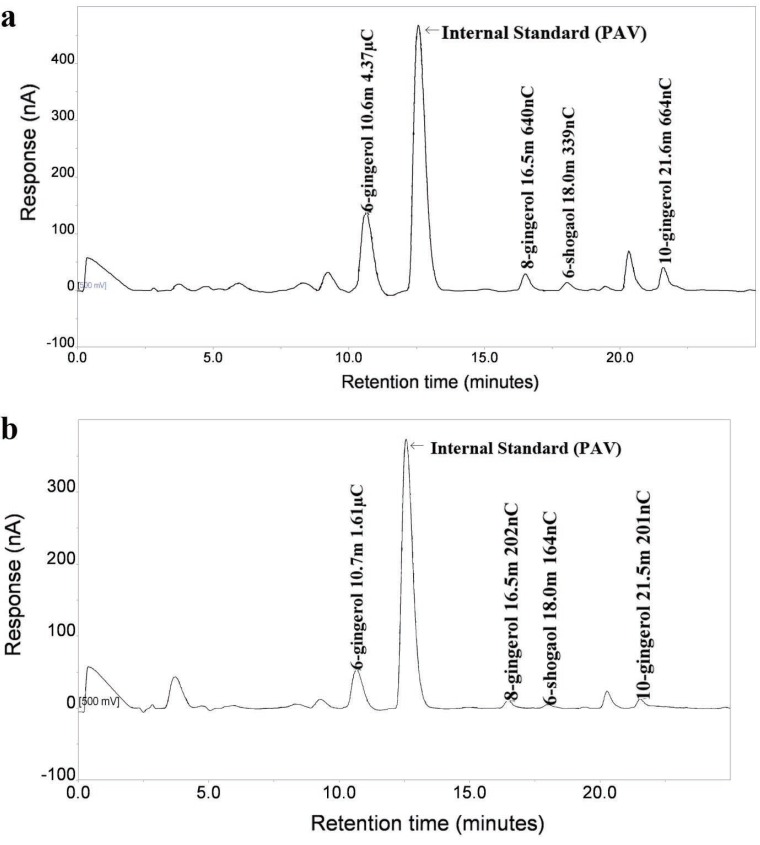
(a), HPLC chromatogram with electrochemical detection (500 mV) obtained from extraction of human plasma 60 minutes after ingesting 2.0 g of ginger extract that was standardized to contain 5.0% gingerols. The mean concentrations, ± SDs and CV of the ginger analytes 60 minutes after dosing were 1.06 ± 0.33 μg/mL for 6-gingerol (CV=30.9%), 0.27 ± 0.15 μg/mL for 8-gingerol (CV=57.4%), 0.46 ± 0.18 μg/mL for 10-gingerol (CV=38.6%) and 0.20 ± 0.12 μg/mL for 6-shogaol (CV=61.8%); (b), HPLC chromatogram with electrochemical detection (500 mV) obtained from extraction of human plasma 30 minutes after ingesting 2.0 g of ginger extract that was standardized to contain 5.0% gingerols. The mean concentrations, ± SDs and CV at 30 minutes were 0.39 ± 0.19 μg/mL for 6-gingerol (CV=48.0%), 0.07 ± 0.05 μg/mL for 8-gingerol (CV=71.3%), 0.13 ± 0.17 μg/mL for 10-gingerol (CV=134.8%) and 0.04 ± 0.03 μg/mL for 6-shogaol (CV=84.1%).

Figures 2a and 2b illustrate the HPLC chromatogram obtained after the extraction of one the study participant’s plasma 60 and 30 minutes after ingesting the ginger capsule. The 30 minute time point was when ginger analytes were first detectable and the 60 minute time point is when the peak concentration of the ginger analytes occurred. Therefore, the figures (Figure 2) demonstrate an example of both high and low concentrations of ginger analytes in plasma.

Before quantifying ginger in plasma, it was necessary to incubate plasma aliquots (500 μL) for one hour at 37°C with 0.1 M, pH5.0) using the method of Asai *et al* (19, 20). The mean concentrations, SDs and CV of the ginger analytes 60 minutes after dosing are shown in Figures 2.

## DISCUSSION

Interpreting the results of phase II/III clinical studies using ginger root for various illnesses is difficult, given the lack of information about the absorption, distribution, excretion and metabolism of important ginger root constituents. Currently, it is unclear if negative study results and modest effect sizes in the majority of the clinical trials of ginger were due to lack of efficacy of the plant or plant extract, inadequate dose or lack of systemic absorption. Consequently, a valid method of detecting major ginger analytes in serum is needed.

A number of analytical methods, e.g., HPLC/ESI-MS/MS, have been developed for detecting and quantifying ginger analytes in methanol solutions, raw plant material, dietary supplements, and spices (20). These methods with the exception of the rodent work by Nakazawa *et al.* (21) were developed as tools to ensure batch-to-batch reproducibility and stability of ginger products, and not to detect ginger constituents in biosample matrices, such as blood.

Several HPLC techniques have been used to quantify 6-gingerol and zingerone in rodents’ bile, urine and plasma (13, 14, 21). These HPLC techniques have neither systematically validated nor have they been replicated for human tissue. The HPLC-based assay method reported by Nakazawa and colleagues (21) requires an analytical run time of 70 minutes and detects two ginger analytes, zingerone and 6-gingerol. No analytical methods have been reported for the other ginger analytes 6-shogaol, 8-gingerol and 10-gingerol in human biosample matrices, and these ginger forms are those found commonly in dried roots, which are most often used as dietary supplements.

Previously, reported HPLC techniques for 6-ginerol had good reproducibility over the concentration range of 0.20 to 40 μg/mL using UV detection (13). The HPLC method reported here, uses electrochemical detection, which can help confirm peak identity, and has good reproducibility over the range of 0.10 to 5.00 μg/mL. The limit of detection is 75 ng/mL or less for gingerols and a limit of quantification of 100 ng/mL (except for 10-gingerol where the limit of quantification is 250 ng/mL). In human plasma, as shown in Figures 2, the concentrations of some ginger analytes were below 200 ng/mL after ingestion of 2.0 grams of a ginger preparation indicating the need for sensitive analytical methods.

In summary, this HPLC technique allows for detection of 6-, 8-, 10-gingerol and 6-shogaol in human plasma. The limit of detection for all four ginger analytes is 75 ng/mL or less and will allow for sensitive pharmacokinetic analyses of ginger analytes in humans. The method allows for the detection of all four of ginger’s pungent constituents simultaneously in a relatively short run time of 25 minutes. This method should be useful for determining plasma levels of 6-, 8-, 10-gingerol and 6-shogaol in phase I clinical trials.
